# Development and Validation of a HPLC-ESI-MS/MS Method for Simultaneous Quantification of Fourteen Alkaloids in Mouse Plasma after Oral Administration of the Extract of *Corydalis yanhusuo* Tuber: Application to Pharmacokinetic Study

**DOI:** 10.3390/molecules23040714

**Published:** 2018-03-21

**Authors:** Weijuan Du, Lisha Jin, Liping Li, Wei Wang, Su Zeng, Huidi Jiang, Hui Zhou

**Affiliations:** College of Pharmaceutical Sciences, Zhejiang University, Hangzhou 310058, China; 21419060@zju.edu.cn (W.D.); jinlisha1211@163.com (L.J.); lillylp@zju.edu.cn (L.L.); ww0912@zju.edu.cn (W.W.); zengsu@zju.edu.cn (S.Z.); hdjiang@zju.edu.cn (H.J.)

**Keywords:** alkaloids, *Corydalis yanhusuo* extract, pharmacokinetics, HPLC-ESI-MS/MS, oxoglaucine

## Abstract

The tuber of *Corydalis yanhusuo* is a famous traditional Chinese medicine and found to have potent pharmacological effects, such as antinociceptive, antitumor, antibacterial, anti-inflammatory, and anti-depressive activities. Although there are several methods to be developed for the analysis and detection of the bioactive ingredients’ alkaloids, so far, only few prominent alkaloids could be quantified, and in vitro and in vivo changes of comprehensive alkaloids after oral administration are still little known. In this study, we first developed a simple and sensitive high-performance liquid chromatography-electrospray ionization-tandem mass spectrometry (HPLC-ESI-MS/MS) method to quantify the comprehensive alkaloids of extracts of *C. yanhusuo* in mouse plasma, using nitidine chloride as an internal standard. As results, at least fourteen alkaloids, including an aporphine (oxoglaucine), a protopine (protopine), five tertiary alkaloids (corydaline, tetrahydroberberine, tetrahydropalmatine, tetrahydrocolumbamine, and tetrahydrocoptisine) and seven quaternary alkaloids (columbamine, palmatine, berberine, epiberberine, coptisine, jatrorrhizine, and dehydrocorydaline) could be well quantified simultaneously in mouse plasma. The lower limits of quantification were greater than, or equal to, 0.67 ng/mL, and the average matrix effects ranged from 96.4% to 114.3%. The mean extraction recoveries of quality control samples were over 71.40%, and the precision and accuracy were within the acceptable limits. All the analytes were shown to be stable under different storage conditions. Then the established method was successfully applied to investigate the pharmacokinetics of these alkaloids after oral administration of the extract of *Corydalis yanhusuo* in mice. To the best of our knowledge, this is the first document to report the comprehensive and simultaneous analyses of alkaloids of *C. yanhusuo* in mouse plasma. It was efficient and useful for comprehensive pharmacokinetic and metabolomic analyses of these complex alkaloids after drug administration.

## 1. Introduction

The dried tuber of *Corydalis yanhusuo* W. T. Wang (Corydalis Rhizoma, or Yuanhu in Chinese) is a famous traditional Chinese medicine (TCM) and widely used for blood activation, moving ‘Qi’ (vital energy), and pain relief for thousands of years in China. It is well known that the main active ingredients of the extract of *C. yanhusuo* are the alkaloids, which possess potent pharmacological effects, such as antinociceptive, antitumor, antibacterial, anti-inflammatory, and anti-depressive activities. For example, tetrahydropalmatine, corydaline, and protopine could enter the central nervous system through the blood-brain barrier, and play the role of analgesic, sedative hypnotic, and anxiolytic effects [1,2,3,4]. Oxoglaucine, berberrabine, berberine, coptisine, palmatine, columbamine, and jatrorrhizine were reported to have antitumor activity [5,6,7], while berberine, palmatine and epiberberine were found to have antibacterial activity [8,9]. In addition, tetrahydropalmatine, palmatine, berberine, dehydrocorydaline, tetrahydroberberine, and tetrahydrocolumbamine have cardioprotective effects [10,11,12].

In view of these various and potent pharmacological activities of alkaloid extracts of *C. yanhusuo*, there are increasing interests on the analyses and identification of these active alkaloid ingredients in vitro and in vivo in order to understand the possible pharmacological effects. Recent studies indicated that protopine [13,14,15,16,17], tertiary alkaloids [18,19,20,21], and quaternary alkaloids [22,23,24,25] could be detected and measured through liquid chromatography with UV or MS. These methods seemed rather efficient and sensitive, however, so far only a few prominent alkaloids could be accurately quantified in each HPLC-MS analysis. It was still difficult to determine in vitro and in vivo changes of comprehensive alkaloids after oral administration.

Therefore, the major purpose of this study was to develop a rapid and sensitive HPLC-ESI-MS/MS method for the simultaneous quantification of these alkaloids in mouse plasma. Meanwhile, we also applied the developed method to the pharmacokinetic study of fourteen alkaloids after oral administration of *Corydalis yanhusuo* extract in mice. Whole results indicated that the developed method was efficient and useful for comprehensive pharmacokinetic analyses of these complex alkaloids, which might also be extended to analyze full metabolomics after drug administration. 

## 2. Experimental

### 2.1. Chemicals and Reagents

**Figure 1:**
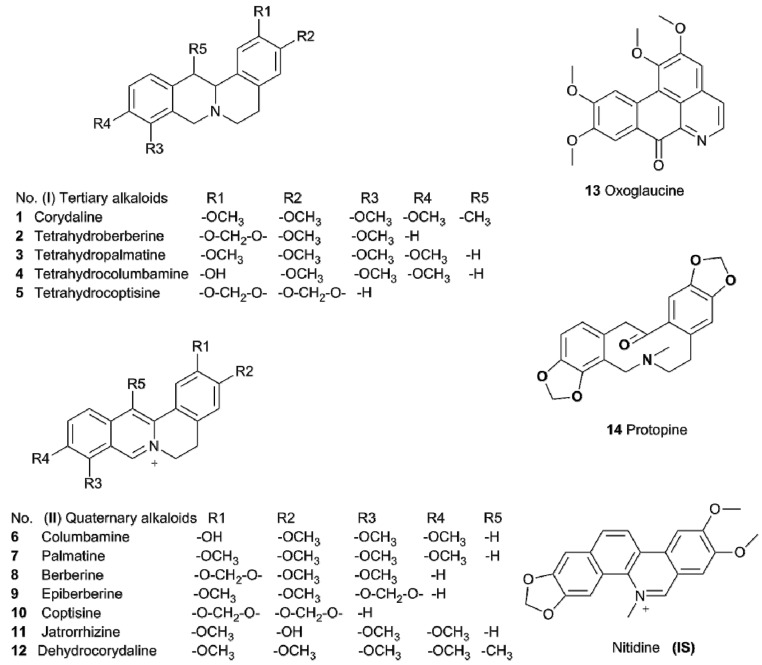
The chemical structures of the fourteen alkaloids and internal standard (IS).

The extract of *Corydalis yanhusuo* was kindly provided by Zhejiang Conba Pharmaceutical Co., Ltd. (Hangzhou, China). Tetrahydropalmatine, jatrorrhizine, and epiberberine were purchased from Aladdin Industrial Corporation (Shanghai, China). Oxoglaucin and columbamine were from Shanghai Winherb Medical Technology Co., Ltd. (Shanghai, China). Nitidine chloride (IS), coptisine, tetrahydroberberine, tetrahydrocoptisine, and palmatine were obtained from Nanjing Zelang Medical Technology Co., Ltd. (Nanjing, China). Berberine, tetrahydrocolumbamine, corydaline, dehydrocorydaline, and protopine were from Nanjing Guangrun Biotechnology Co., Ltd. (Nanjing, China). The purity of all the reference standards was higher than 98%. Dimethyl sulfoxide (DMSO) was purchased from Sigma-Aldrich (St. Louis, MO, USA). Acetonitrile was obtained from Tedia (Fairfield, TX, USA). Formic acid was bought from Anaqua Chemicals Supply (Houston, TX, USA). All other chemicals were of analytical grade and were obtained commercially. The chemical structures of the alkaloids are shown in Figure 1.

### 2.2. HPLC–MS/MS Instrumentation and Chromatography Conditions

The analyses were performed with the 1290 Infinity HPLC system, which was coupled to a 6460 triple quadrupole mass spectrometer (Agilent Technologies, Santa Clara, CA, USA) with an electrospray ionization (ESI) source. MassHunter Workstation software (version B.06.00 SP01, Agilent Technologies, Santa Clara, CA, USA) was used.

Chromatographic separation was performed on a ZORBAX Eclipse XDB-C18 column (2.1 × 100 mm, 3.5 μm, Agilent Technologies) maintained at 35 °C at a flow rate of 0.3 mL/min. The gradient elution was used with solvents consisting of 0.1% formic acid in water (A) and acetonitrile (B). The LC gradient condition was optimized as follows: 20–26% of B (0–2.5 min); 26–40% of B (2.5–5 min); 40–80% of B (5–5.4 min); 80% of B (5.4–5.9 min); 20% of B (6–7.5 min).

Mass spectrometric analysis was performed in positive ion mode. The conditions of MS analysis were designed and set as follows: capillary voltage 3.5 kV, Nozzle voltage 300 V, sheath gas (nitrogen) heater temperature 350 °C and drying gas (nitrogen) temperature 325 °C. The settings included sheath gas at 11 L/min, drying gas at 6 L/min, nebulizer set at 45 psi, and a dwell time of 20 ms. Quantification was obtained using different multiple reaction monitoring (MRM) transitions. A summary of detection MS/MS parameters is shown in Table 1. 

### 2.3. Preparation of Calibration Standard and Quality Control (QC) Samples

The primary stock solution of each analyte and IS (concentrations ranging from 10 to 100 mmol/L) was made in DMSO based on the solubility of individual alkaloids. A stock solution (1 mmol/L) of each analyte was prepared by diluting the primary stock solution with DMSO. The IS working solution of 10 μg/mL was prepared by diluting the IS primary stock solution (10 mmol/L) with DMSO. A series of standard mixture working solutions (Table 2) was used for the fresh preparation of calibration samples and quality control samples using both heparinized and blank mouse plasma before each analytical run. All the stock and working standard solutions were sub-packed and stored below −20 °C until use. 

### 2.4. Sample Preparation

Plasma samples were removed from −20 °C storage and thawed at room temperature. Then, 50 μL of plasma was vortex mixed with 500 μL of acetonitrile/acetone (90:10, *v*/*v*) containing IS solution (0.5 ng/mL) in a 1.5-mL Eppendorf tube for 6 min. The mixture was then centrifuged at 13,000 rpm for 15 min. Then, 500 μL of supernatant was transferred to another Eppendorf tube and volatilized to dryness at 40 °C with a vacuum concentrator system (LABCONCO, Kansas City, MO, USA). The residue was re-constituted in 100 μL of acetonitrile/water (30:70, *v*/*v*) containing 0.1% formic acid by vortex for 6 min, and centrifuged at 13,000 rpm for 15 min. The supernatant was transferred to an auto-sampler vial, and an aliquot of 5 μL was injected into the HPLC–MS/MS system for simultaneous analysis.

### 2.5. Method Validation

#### 2.5.1. Selectivity and Specificity

Specificity was evaluated by analyzing blank plasma samples from six mice to investigate the potential endogenous interference at retention times of analytes and IS. A comparison study was performed on chromatograms of blank plasma, plasma samples spiked with the 14 analytes, and IS, as well as plasma samples after an oral dose.

#### 2.5.2. Linearity and Lower Limits of Quantification (LLOQ)

Calibration curves were prepared by assaying standard calibration samples at eight concentration levels. The linearity of each calibration curve was determined by plotting the peak area ratio (Y) of fourteen analytes to IS versus the nominal concentration (χ) of analytes with weighted (1/χ^2^) least square linear regression. 

#### 2.5.3. Precision and Accuracy

The intra- and inter-batch precision, which was evaluated by relative standard deviation (RSD), was determined through quantifying three concentrations levels of QC samples (five samples for LLOQ level) on the same analytical run and for three different analytical runs. Accuracy was evaluated as the relative error (RE) using the formula (1 − average measured mean value/the nominal value) × 100%. 

#### 2.5.4. Extraction Recovery and Matrix Effect

The extraction recoveries of analytes at three QC levels were determined by comparing the peak areas obtained from five extracted QC samples with five unextracted samples [26]. The absolute matrix effects of analytes and IS were evaluated by comparing the peak areas obtained from samples in which the extracted blank mice plasma were spiked with standard solutions compared to the pure reference standard solutions at the same concentration. The relative matrix effects were the ratio of analytes’ absolute matrix effects to IS absolute matrix effects.

#### 2.5.5. Stability Experiment and Dilution Integrity

The stability was assessed using QC samples at low and high concentrations, which were freshly prepared and immediately mixed by vortex for 3 min followed by storage at −20 °C for ten days and for three months to evaluate the short-term and long-term frozen stability. The short-term stability was determined by thawing the QC samples at room temperature (approximately 25 °C) for 8 h, which exceeded the routine preparation time for the samples. The post-preparation stability was tested by measuring the extracted QC samples stored in the auto-sampler (10 °C) for 12 h. The freeze and thaw stability were determined using QC samples after one, two, and three freeze (−20 °C)–thaw (room temperature) cycles. 

The dilution integrity of samples was tested by spiking blank mouse plasma with standard mixture working solutions at a high concentration and diluting the obtained samples with blank mouse plasma at a ratio of 1:10 and 1:20. Five replicates were analyzed. Carry-over was assessed by directly injecting three blank solvents of acetonitrile samples after injecting the upper limit of quantification (ULOQ) samples.

### 2.6. Pharmacokinetic Study

The studies were approved by the Institutional Animal Care and Use Committee of Zhejiang University Medical Center (ZJU2015-523-01). One hundred and twelve male ICR mice, 18–25 g, were obtained from the Experimental Animal Center of the Zhejiang Academy of Medical Sciences (Hangzhou, China). The animals were acclimatized to the facilities for a week and then fasted with free access to water overnight (approximately 12 h) before the experiment. The *Corydalis yanhusuo* extract was suspended in 0.5% carboxymethyl cellulose sodium (CMC-Na) aqueous solution and was given to the mice intragastrically (1.25 g extract/kg body weight), and HPLC analyses indicated that the contents of 14 marker compounds in the extract are listed as follows: corydaline, 17.01 mg/g; tetrahydroberberine, 5.966 mg/g; tetrahydropalmatine, 8.32 mg/g; tetrahydrocolumbamine, 8.79 mg/g; tetrahydrocoptisine, 4.197 mg/g; columbamine, 2.699 mg/g; palmatine, 4.566 mg/g; berberine, 1.113 mg/g; epiberberine, 0.1316 mg/g; coptisine, 50.35 mg/g; jatrorrhizine, 2.428 mg/g; dehydrocorydaline, 14.33 mg/g; oxoglaucine, 0.2727 mg/g; protopine 4.529 mg/g. After treatment at 0, 5, 10, 15, 30, 45, 60, 120, 180, 240, 360, 480, 720, and 1440 min, approximately 300 μL of blood was collected from the oculi chorioideae vein and put into heparinized 1.5-mL Eppendorf tubes at predetermined time points (*n* = 8/group). Subsequently, the plasma was centrifuged at 6000 rpm for 10 min to separate the plasma, and then the plasma was transferred to clean 1.5-mL Eppendorf tubes and frozen at −20 °C until analysis. 

### 2.7. Date Analysis

The pharmacokinetic parameters of fourteen alkaloids were calculated with the non-compartmental analysis of plasma concentration vs. time data using DAS 2.0 software (BioGuider Co., Shanghai, China), and the mean plasma concentration-time curves were plotted using GraphPad Prism (Version 6.01, GraphPad Software, La Jolla, CA, USA) software.

## 3. Results and Discussion

### 3.1. Method Development

#### 3.1.1. Optimization of HPLC–MS/MS Conditions

ESI-MS was used to analyze the mass spectra of the fourteen alkaloids and IS is shown in Supplemental Figure S1.

The mobile phase played a key role in the acquisition of good chromatographic behavior and appropriate ionization. We found that acetonitrile, as the organic phase, resulted in lower background noise, lower column pressure, and better peak shape than methanol. To obtain relatively short retention times and high detection sensitivity, different amounts of formic acid were added to the aqueous phases, adjusting to pH 3 (0.1% formic acid), pH 4, pH 5, pH 5.5, and pH 6 with a linear gradient elution. When pH = 3, the retention time of alkaloids was the shortest with high detection sensitivity because excessive formic acid provided enough protons for promoting ionization. However, columbamine and jatrorrhizine as a pair of isomeric compounds could not be separated well by HPLC on an X-Bridge C18 column (4.6 × 150 mm, 3.5 µm, Waters, Milford, MA, USA). Therefore, a ZORBAX Eclipse XDB-C18 column (2.1 × 100 mm, 3.5 μm, Agilent) with high performance over a wide pH range (2–9) was tried at different gradient elutions and at different flow rates until isomers in two groups (columbamine and jatrorrhizine, and berberine and epiberberine) could be separated. It is noteworthy that a high proportion of the organic phase (e.g., 80% acetonitrile) should be kept for a while in setting the posterior program of LC gradient elution, which was helpful for cleaning the column. Finally, a ZORBAX Eclipse XDB-C18 column using acetonitrile-water (0.1% formic acid) as a mobile phase system with a gradient procedure at a flow rate of 0.3 mL/min was completed within 7.5 min.

#### 3.1.2. Optimization of Sample Preparation

To eliminate interference from the sample matrix and achieve satisfactory recovery, liquid–liquid extraction with ethyl acetate and a protein precipitation with different precipitants was compared during sample preparation. We found that the extraction recoveries of tertiary alkaloids were high with a low level of endogenous interference using LLE for sample preparation in mouse plasma, whereas the extraction recoveries of certain quaternary alkaloids, such as columbamine, berberine, palmatine, and dehydrocorydaline, were less than 50% no matter how the pH value was regulated. Precipitants, such as acetonitrile, methanol, acetone, and a mixture of different proportions of acetonitrile and acetone, were tried, of which acetonitrile/acetone (90:10, *v*/*v*), was the most efficient extraction solvent. We chose a quaternary alkaloid, nitidine chloride, as an IS, because it was stable and similar to the analyte signal in most ways, which improved the precision of quantitative analysis. The medicinal concentrations in biological samples were low, so enrichment was needed. In addition, re-constituted solvents were tested in acetonitrile and water at different mixing ratios, and we found that acetonitrile/water (30:70, *v*/*v*) containing 0.1% formic acid produced a good peak shape.

### 3.2. Method Validation

#### 3.2.1. Specificity and Selectivity

**Figure 2:**
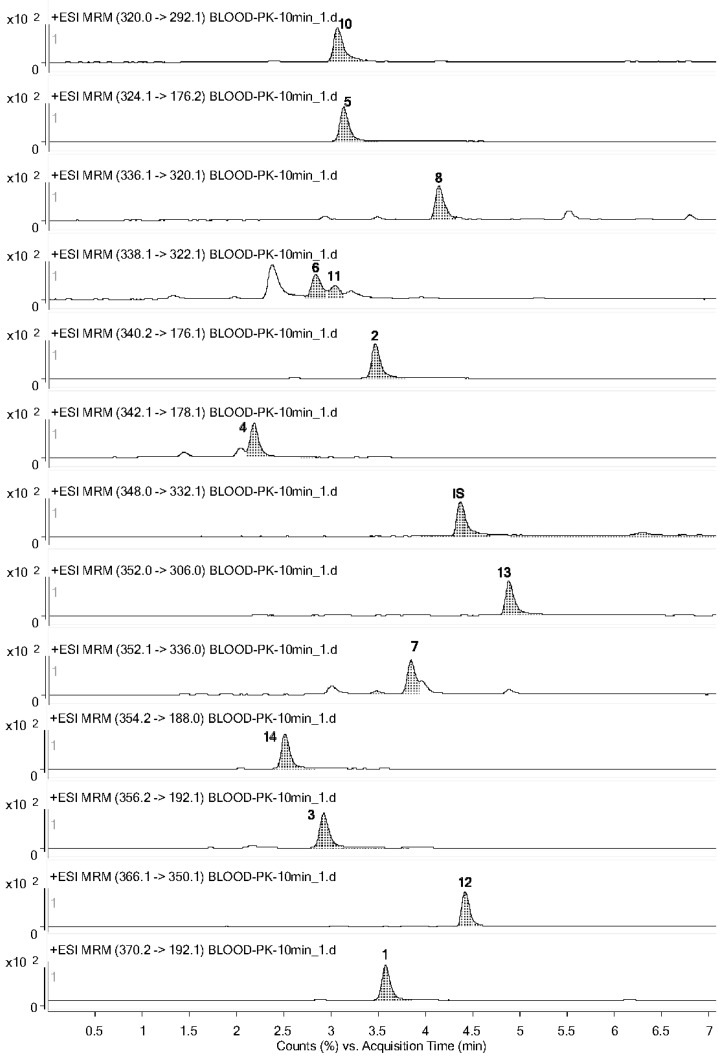
Representative MRM chromatograms of plasma samples at 10 min following oral administration of *Corydalis yanhusuo* extract.

The representative chromatograms of (1) blank plasma, (2) blank plasma spiked with the standard solutions, and (3) plasma samples obtained following oral administration of *Corydalis yanhusuo* extract in ICR mice are shown in Figure 2 and Supplementary Figure S2. Under the established chromatography conditions, all 14 analytes and IS could be separated well from each other. The responses of endogenous interference peaks in the blank plasma were less than 20% of the LLOQ and 5% of the IS at the retention positions of all 14 analytes, demonstrating that the method was specific. 

#### 3.2.2. Linearity and Sensitivity

As shown in Table 2, all the calibration curves demonstrated good linearity with every correlation coefficient (r) over 0.996. The LLOQ of all the alkaloids achieved a signal-to-noise (S/N) ratio higher than 10, as well as precision and accuracy less than 20% and within ±20%, respectively. This sensitivity has proven useful for measuring the trace concentration of analytes in plasma.

#### 3.2.3. Precision and Accuracy

As shown in Table 3, the intra- and inter-day precision values (% RSD) were both less than 13%, whereas the accuracy values (% RE) ranged from −9.16–11.09% for each QC level of the analytes. These results, which were all within the acceptable criteria for accuracy and precision, indicate that the method is reliable and reproducible for the measuring fourteen alkaloids in mouse plasma.

#### 3.2.4. Extraction Recovery and Matrix Effect

As shown in Table 4, the average matrix effects at three level QC samples ranged from 96.40% to 114.3% with RSD values within 5.8%, while the average matrix effects of IS were 99.5%, which suggests that ion suppression and enhancement from plasma are consistent for the samples and IS. The mean extraction recoveries of all the analytes, including IS, were over 71.40%, and the RSD values were less than 11.0%. 

#### 3.2.5. Stability and Dilution Integrity

The stability experimental results under different storage conditions are presented in Table 5. The results showed that samples were stable during three months of storage at −20 °C, 8 h at room temperature, 12 h at auto-sampler temperature with an accuracy within ±15%, and precision less than 15%. However, several values of freeze-thaw cycles are larger than 15%, which suggests that we should reduce the number of freeze-thaw times as much as possible, so we sub-packed and stored below −20 °C until use in the experiment.

Dilution integrity results are presented in Table 6. Samples that were diluted 10-fold and 20-fold did not affect the precision and accuracy of the method because the precision (RSD%) ranged from 0.8–8.4%, and the accuracy (RE%) was below 11.4% for all alkaloids that were within the set criteria. The mean carry-over in blank mice plasma samples following the ULOQ sample was lower than 20% of the LLOQ and 5% for the IS, which met the requirements for the EMA guidelines for industry.

### 3.3. Pharmacokinetic Study

**Figure 3:**
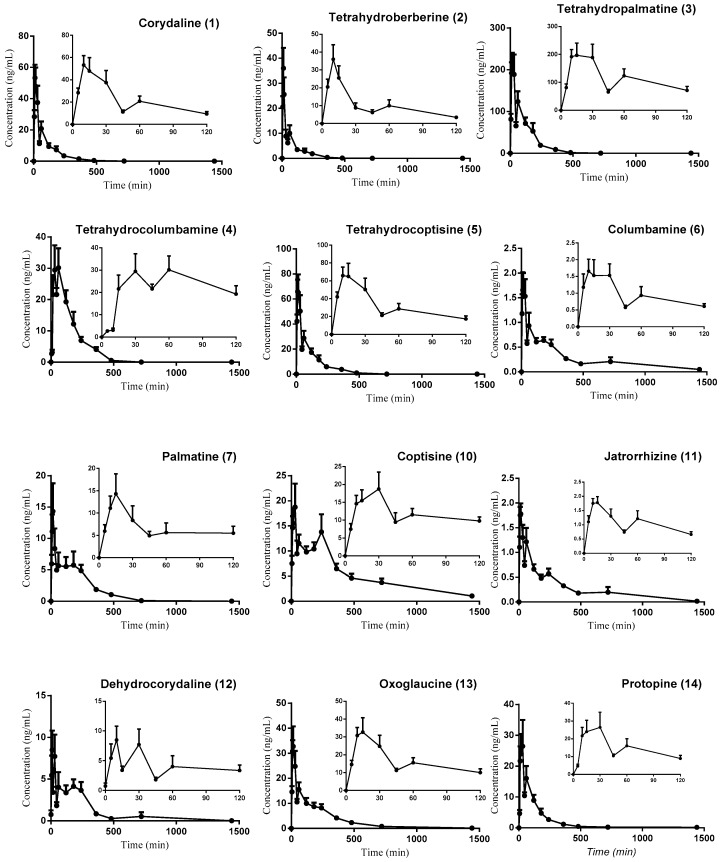
Mean plasma concentration-time profiles of twelve alkaloids after oral administration of *Corydalis yanhusuo* extract in male ICR mice (1.25 g/kg) (mean ± SEM).

The validated HPLC-ESI-MS/MS method was successfully applied to the pharmacokinetic study and simultaneous measurement of corydaline, columbamine, palmatine, oxoglaucine, protopine, tetrahydroberberine, tetrahydropalmatine, coptisine, jatrorrhizine, tetrahydrocolumbamine, tetrahydrocoptisine, and dehydrocorydaline in mouse plasma for 24 h after oral administration of 1.25 g of *Corydalis yanhusuo* extract per kg of body weight. The typical MRM chromatograms of mouse plasma are shown in Figure 2. The mean plasma concentration–time profiles of twelve alkaloids are shown in Figure 3, and the main pharmacokinetic parameters assessed by noncompartmental analysis are listed in Table 7. The results showed that the plasma concentration-time profiles of berberine and epiberberine could not be acquired because the concentration of these two compounds in most plasma samples was below the LLOQ. The T_max_ values of tetrahydrocolumbamine and coptisine were (35.63 ± 11.16) min and (28.13 ± 9.61) min, respectively, and the T_max_ values of the remaining alkaloids were within 30 min of the fast absorption rate. It was shown from pharmacokinetics parameters that the C_max_, AUC_0−t_, and AUC_0−∞_ of tetrahydropalmatine were the highest of all the alkaloids. However, the T_1/2z_ of tetrahydropalmatine was the shortest of all the alkaloids, indicating that the elimination rate of tetrahydropalmatine was the fastest.

## 4. Conclusions

In this study, a rapid and sensitive HPLC-ESI-MS/MS method has been developed and validated for simultaneous analyses of at least 14 alkaloids, including an aporphine (oxoglaucine), a protopine (protopine), five tertiary alkaloids, and seven quaternary alkaloids within 7.5 min in mouse plasma. This method was successfully applied to the pharmacokinetic study of twelve alkaloids after oral administration of *Corydalis yanhusuo* extract in mice. This is the first document to report the comprehensive and simultaneous analyses of alkaloids of *C. yanhusuo* in mouse plasma. It was efficient and useful for comprehensive pharmacokinetic and metabolomic analyses of these complex alkaloids after drug administration.

## Figures and Tables

**Table 1:** MRM transitions and parameters of the fourteen alkaloids and IS.

No.	Alkaloids	Ret. Time (min)	MRM Transition Precursor Ion > Product Ion	Fragmentor Voltage (V)	Collision Energy (eV)
**1**	Corydaline	3.67	370.2 > 192.1	150	27
**2**	Tetrahydroberberine	3.54	340.2 > 176.1	150	27
**3**	Tetrahydropalmatine	3.00	356.2 > 192.1	150	29
**4**	Tetrahydrocolumbamine	2.08	342.1 > 178.1	150	27
**5**	Tetrahydrocoptisine	3.19	324.1 > 176.2	150	24
**6**	Columbamine	2.90	338.1 > 322.1	150	27
**7**	Palmatine	4.04	352.3 > 336.2	150	30
**8**	Berberine	4.21	336.1 > 320.1	150	30
**9**	Epiberberine	2.98	336.1 > 320.1	150	30
**10**	Coptisine	3.12	320.0 > 292.1	150	30
**11**	Jatrorrhizine	3.04	338.1 > 322.1	150	27
**12**	Dehydrocorydaline	4.49	366.1 > 350.1	150	30
**13**	Oxoglaucine	4.89	352.3 > 306.0	150	28
**14**	Protopine	2.58	354.2 > 188.0	150	30
IS	Nitidine chloride	4.43	348.1 > 332.0	210	35

**Table 2:** The regress equations, linear range, and LLOQs of the fourteen alkaloids.

No.	Alkaloids	Linear Regression Equation	Range (ng/mL)	Correlation Coeffiicient (r)	LLOQ (ng/mL)
**1**	Corydaline	Y = 0.139805χ − 0.006704	0.74–73.89	0.998	0.74
**2**	Tetrahydroberberine	Y = 0.097085χ − 0.004676	0.68–67.88	0.998	0.68
**3**	Tetrahydropalmatine	Y = 0.301153χ + 0.199059	1.78–177.7	0.996	1.78
**4**	Tetrahydrocolumbamine	Y = 0.216374χ − 0.021908	1.71–170.7	0.998	1.71
**5**	Tetrahydrocoptisine	Y = 0.177838χ − 0.044206	1.62–161.7	0.997	1.62
**6**	Columbamine	Y = 0.089586χ + 0.011028	0.68–67.68	0.999	0.68
**7**	Palmatine	Y = 0.120359χ + 0.052267	0.71–70.48	0.998	0.71
**8**	Berberine	Y = 0.125996χ + 0.005547	0.67–67.27	0.999	0.67
**9**	Epiberberine	Y = 0.072013χ − 0.003910	0.67–67.27	0.999	0.67
**10**	Coptisine	Y = 0.007792χ + 0.11179	3.20–320.3	0.998	3.20
**11**	Jatrorrhizine	Y = 0.085938χ − 0.008861	0.68–67.68	0.999	0.68
**12**	Dehydrocorydaline	Y = 0.106311χ + 0.107424	1.83–183.2	0.998	1.83
**13**	Oxoglaucine	Y = 0.113817χ + 0.031077	0.70–70.27	0.998	0.70
**14**	Protopine	Y = 0.106329χ + 0.007831	0.71–70.67	0.999	0.71

**Table 3:** Intra-day and inter-day precisions and accuracies of the fourteen alkaloids from the assay samples (mean ± SD).

Alkaloids	Nominal Conc. (ng/mL)	Intra-Day Batch (*n* = 5)			Inter-Day Batch (*n* = 9)		
		Observed Conc. (ng/mL)	Precision (%, RSD)	Accuracy (%, RE)	Observed Conc. (ng/mL)	Precision (%, RSD)	Accuracy (%, RE)
Corydaline	0.74	0.73 ± 0.023	3.1	−1.5	0.75 ± 0.012	1.6	1.1
	1.85	1.86 ± 0.096	5.2	0.5	1.82 ± 0.059	3.2	−1.6
	9.24	9.59 ± 0.21	2.2	3.8	9.39 ± 0.31	3.3	1.6
	55.42	53.27 ± 1.36	2.5	−3.9	55.9 ± 2.01	3.6	0.9
Tetrahydroberberine	0.68	0.68 ± 0.017	2.5	0.7	0.68 ± 0.019	2.9	−0.6
	1.70	1.72 ± 0.039	2.2	1.3	1.68 ± 0.033	2.0	−1.2
	8.48	8.82 ± 0.16	1.8	4.0	8.68 ± 0.35	4.0	2.3
	50.91	48.51 ± 1.18	2.4	−4.7	51.32 ± 1.97	3.8	0.8
Tetrahydropalmatine	1.78	1.78 ± 0.099	5.5	0.2	1.78 ± 0.024	1.4	0.2
	3.55	3.66 ± 0.14	3.9	3.0	3.56 ± 0.09	2.5	0.2
	17.77	19.15 ± 0.28	1.5	7.7	18.7 ± 0.60	3.2	5.2
	142.17	129.2 ± 2.92	2.3	−9.2	137.2 ± 5.75	4.2	−3.5
Tetrahydrocolumbamine	1.71	1.72 ± 0.038	2.2	0.9	1.72 ± 0.036	2.1	0.9
	3.41	3.61 ± 0.036	1.0	5.6	3.40 ± 0.14	4.0	−0.3
	17.07	17.11 ± 0.83	4.8	0.3	17.34 ± 0.27	1.6	1.6
	136.6	133.1 ± 2.52	1.9	−2.5	134.1 ± 6.47	4.8	−1.8
Tetrahydrocoptisine	1.62	1.48 ± 0.050	3.4	−8.8	1.59 ± 0.067	4.2	−1.6
	3.23	3.42 ± 0.12	3.5	5.6	3.16 ± 0.11	3.5	−2.2
	16.17	15.74 ± 0.98	6.2	−2.6	16.33 ± 0.31	1.9	1.0
	129.3	130.4 ± 3.11	2.4	0.8	127.7 ± 5.12	4.0	−1.3
Columbamine	0.68	0.65 ± 0.011	1.6	−4.1	0.67 ± 0.013	2.0	−0.4
	1.69	1.75 ± 0.056	3.2	3.5	1.66 ± 0.043	2.6	−1.7
	8.46	8.89 ± 0.25	2.8	5.0	8.76 ± 0.41	4.6	3.6
	50.76	49.29 ± 1.59	3.2	−2.9	51.63 ± 1.95	3.8	1.7
Palmatine	0.70	0.69 ± 0.032	−1.9	4.6	0.71 ± 0.015	2.1	0.4
	1.76	1.90 ± 0.16	8.2	7.9	1.78 ± 0.059	3.3	0.9
	8.81	9.26 ± 0.20	2.1	5.2	8.95 ± 0.22	2.5	1.6
	52.86	50.70 ± 1.36	2.7	−4.1	52.6 ± 1.50	2.8	−0.5
Berberine	0.67	0.67 ± 0.014	2.1	−0.3	0.68 ± 0.013	2.0	0.6
	1.68	1.78 ± 0.12	6.9	5.9	1.68 ± 0.027	1.6	0.1
	8.41	8.77 ± 0.25	2.9	4.2	8.62 ± 0.24	2.8	2.5
	50.45	48.34 ± 1.20	2.5	−4.2	50.23 ± 1.30	2.6	−0.4
Epiberberine	0.67	0.68 ± 0.036	5.3	1.1	0.69 ± 0.022	3.2	2.3
	1.68	1.71 ± 0.088	5.2	1.9	1.66 ± 0.051	3.0	−1.0
	8.41	8.86 ± 0.19	2.1	5.3	8.57 ± 0.26	3.0	2.0
	50.45	48.76 ± 1.49	3.1	−3.4	50.19 ± 1.31	2.6	−0.5
Coptisine	3.20	2.83 ± 0.18	6.5	−11.5	3.16 ± 0.18	5.7	−1.5
	6.41	6.87 ± 0.87	12.6	7.3	6.35 ± 0.31	4.8	−0.9
	32.03	35.58 ± 3.82	10.7	11.1	30.97 ± 0.99	3.2	−3.3
	256.3	254.7 ± 15.00	5.9	−0.6	256.9 ± 7.23	2.8	0.2
Jatrorrhizine	0.68	0.69 ± 0.021	3.1	1.6	0.70 ± 0.015	2.2	3.6
	1.69	1.79 ± 0.045	2.5	5.9	1.65 ± 0.058	3.5	−2.3
	8.46	8.73 ± 0.33	3.8	3.2	8.54 ± 0.14	1.6	0.9
	50.76	48.82 ± 1.48	3.0	−3.8	51.03 ± 3.41	6.7	0.5
Dehydrocorydaline	1.83	1.76 ± 0.23	12.9	−3.8	1.78 ± 0.11	6.4	−2.8
	3.66	3.85 ± 0.27	6.9	5.0	3.65 ± 0.12	3.3	−0.4
	18.32	17.28 ± 1.39	8.0	−5.7	18.16 ± 0.34	1.9	−0.9
	146.6	144.5 ± 5.45	3.8	−1.4	144.1 ± 2.86	2.0	−1.7
Oxoglaucine	0.70	0.66 ± 0.045	6.8	−5.5	0.69 ± 0.039	5.6	−1.5
	1.76	1.82 ± 0.075	4.1	3.6	1.75 ± 0.021	1.2	−0.1
	8.78	9.57 ± 0.20	2.1	9.0	9.13 ± 0.26	2.9	3.9
	52.7	50.76 ± 1.52	3.0	−3.7	52.83 ± 1.29	2.4	0.2
Protopine	0.71	0.68 ± 0.019	2.7	−3.5	0.71 ± 0.013	1.9	0.9
	1.77	1.80 ± 0.050	2.8	1.7	1.77 ± 0.042	2.4	0.3
	8.83	9.34 ± 0.16	1.7	5.8	9.16 ± 0.32	3.5	3.7
	53.01	51.00 ± 1.40	2.7	−3.8	53.36 ± 1.94	3.6	0.7

**Table 4:** Matrix effects and extraction recoveries of the fourteen alkaloids in mouse plasma.

Alkaloids	Conc. (ng/mL)	Recovery (%)		Absolute Matrix Effect (%)	
		(Mean ± SD, *n* = 5)	RSD	(Mean ± SD, *n* = 6)	RSD
Corydaline (**1**)	1.85	93.73 ± 3.12	3.3	102.5 ± 1.55	1.5
	9.24	92.12 ± 3.30	3.6	102.2 ± 2.39	2.3
	55.42	92.57 ± 2.48	2.7	98.28 ± 2.45	2.5
Tetrahydroberberine (**2**)	1.70	95.83 ± 2.19	2.3	103.0 ± 2.13	2.1
	8.48	92.39 ± 3.56	3.8	100.5 ± 2.24	2.2
	50.91	93.16 ± 2.8	3.0	96.40 ± 2.95	3.1
Tetrahydropalmatine (**3**)	3.55	94.18 ± 2.37	2.5	106.6 ± 2.32	2.2
	17.77	91.74 ± 2.77	3.0	103.8 ± 2.38	2.3
	142.2	108.0 ± 1.97	1.8	100.4 ± 2.79	2.8
Tetrahydrocolumbamine (**4**)	3.41	83.00 ± 2.55	3.1	106.5 ± 2.08	2.0
	17.07	86.30 ± 1.53	1.8	106.1 ± 1.80	1.7
	136.6	86.38 ± 1.94	2.2	110.6 ± 2.86	2.6
Tetrahydrocoptisine (**5**)	3.23	71.73 ± 2.77	3.9	104.2 ± 4.21	4.0
	16.17	76.32 ± 1.15	1.5	102.3 ± 1.18	1.2
	129.3	80.90 ± 4.08	5.0	102.4 ± 4.96	4.8
Columbamine (**6**)	1.69	96.77 ± 5.95	6.2	105.7 ± 2.78	2.6
	8.46	91.23 ± 3.15	3.5	101.7 ± 2.85	2.8
	50.76	92.41 ± 2.55	2.8	98.07 ± 2.64	2.7
Palmatine (**7**)	1.76	95.88 ± 8.92	9.3	108.0 ± 3.28	3.0
	8.81	86.52 ± 3.24	3.8	102.6 ± 2.46	2.4
	52.86	89.90 ± 2.30	2.6	99.70 ± 2.84	2.8
Berberine (**8**)	1.68	97.73 ± 7.28	7.4	102.2 ± 2.28	2.2
	8.41	88.21 ± 4.34	4.9	101.6 ± 2.48	2.4
	50.45	90.29 ± 2.58	2.9	97.93 ± 2.83	2.9
Epiberberine (**9**)	1.68	90.67 ± 3.96	4.4	105.3 ± 3.61	3.4
	8.41	88.14 ± 3.61	4.1	101.7 ± 2.09	2.0
	50.45	90.49 ± 3.22	3.6	97.60 ± 2.82	2.9
Coptisine (**10**)	6.41	96.83 ± 10.61	11.0	107.5 ± 5.78	5.4
	32.03	93.39 ± 9.84	10.5	101.9 ± 1.74	1.7
	256.3	92.03 ± 5.11	5.6	97.06 ± 2.91	3.0
Jatrorrhizine (**11**)	1.69	76.38 ± 2.83	3.7	108.9 ± 2.66	2.4
	8.46	82.54 ± 1.49	1.8	104.6 ± 1.56	1.5
	50.76	72.26 ± 2.30	3.2	108.0 ± 3.71	3.4
Dehydrocorydaline (**12**)	3.66	83.90 ± 3.45	4.1	112.4 ± 3.40	3.0
	18.32	83.51 ± 5.27	6.3	110.9 ± 6.39	5.8
	146.6	86.45 ± 3.14	3.6	109.0 ± 2.40	2.2
Oxoglaucine (**13**)	1.76	111.2 ± 3.08	2.8	109.6 ± 5.53	5.0
	8.78	104.4 ± 2.82	2.7	115.4 ± 5.97	5.2
	52.7	112.3 ± 4.27	3.8	108.4 ± 3.23	3.0
Protopine (**14**)	1.77	94.86 ± 3.04	3.2	105.2 ± 1.11	1.1
	8.83	92.52 ± 2.94	3.2	102.7 ± 2.72	2.6
	53.01	94.86 ± 2.48	2.6	98.65 ± 2.93	3.0
IS	5.00	71.40 ± 2.89	4.0	99.50 ± 3.11	3.1

**Table 5:** The stability of the fourteen alkaloids in ICR mice plasma under different storage conditions.

Alkaloids	Spiked Conc. (ng/mL)	Room Temp. for 8 h		Post-Preparation Stability (10 °C) for 12 h		Frozen for 3 Months (−20 °C)		Three-Freeze–Thaw Cycle	
		Accuracy (%, RE)	Precision (%, RSD)	Accuracy (%, RE)	Precision (%, RSD)	Accuracy (%, RE)	Precision (%, RSD)	Accuracy (%, RE)	Precision (%, RSD)
Corydaline (**1**)	1.85	−9.8	4.9	−4.1	1.0	9.6	9.3	−14.0	8.7
	55.42	−9.4	0.4	7.7	4.5	−2.2	6.6	−7.0	4.3
Tetrahydroberberine (**2**)	1.70	−9.5	2.1	−3.8	1.8	8.9	7.5	−13.2	6.6
	50.91	−10.3	1.2	6.3	4.2	1.5	7.6	−8.8	3.5
Tetrahydropalmatine (**3**)	3.55	−8.8	1.8	−0.1	0.8	6.2	7.0	−13.1	6.8
	142.2	−13.6	1.3	0.5	4.0	−4.1	7.6	1.8	1.6
Tetrahydrocolumbamine (**4**)	3.41	14.2	2.7	1.8	4.4	4.6	3.2	−10.0	1.2
	136.6	8.8	1.5	−3.2	0.7	3.8	1.3	−2.9	3.4
Tetrahydrocoptisine (**5**)	3.23	12.9	1.4	4.8	2.2	5.6	2.6	−11.0	1.5
	129.3	6.6	2.0	−1.9	1.4	4.3	0.6	−7.7	3.9
Columbamine (**6**)	1.69	−7.9	1.9	1.2	0.8	−10.7	1.0	−12.2	5.5
	50.76	−5.6	2.2	8.5	3.3	0.7	6.5	−10.4	3.2
Palmatine (**7**)	1.76	−14.5	3.4	−4.0	2.6	−4.0	5.3	−8.6	8.5
	52.86	−8.6	3.3	5.5	3.9	3.3	5.6	−12.7	3.5
Berberine (**8**)	1.68	3.8	4.3	6.5	0.3	2.3	7.0	−11.7	6.2
	50.45	−9.0	1.5	5.3	3.6	1.7	5.0	−12.1	3.9
Epiberberine (**9**)	1.68	−4.4	2.8	−3.2	2.4	6.5	3.7	−10.7	4.3
	50.45	−7.6	1.5	5.6	2.7	4.4	6.5	−10.4	2.9
Coptisine (**10**)	6.41	4.0	3.5	3.0	0.5	10.9	5.5	−12.9	5.9
	256.3	−6.5	1.1	6.2	4.1	3.2	6.0	−11.4	4.2
Jatrorrhizine (**11**)	1.69	11.4	6.2	−1.1	3.2	2.6	7.3	−3.8	2.5
	50.76	11.3	12.4	−4.1	0.5	7.0	1.4	−15.6	3.7
Dehydrocorydaline (**12**)	3.66	10.6	2.6	−0.8	3.2	7.2	6.0	−19.0	3.3
	146.6	3.4	1.0	−0.2	0.4	0.6	0.8	−2.7	2.8
Oxoglaucine (**13**)	1.76	10.2	3.8	0.2	1.6	8.4	3.7	−6.0	8.9
	52.7	−6.4	1.8	0.1	2.0	−4.1	1.5	−13.2	0.8
Protopine (**14**)	1.77	−6.5	2.6	0.7	0.5	1.4	3.2	−13.1	4.6
	53.01	−5.8	6.3	6.5	3.9	0.5	7.1	−11.1	2.5

**Table 6:** The dilution integrity of the fourteen alkaloids in ICR mice plasma (mean ± SD, *n* = 5).

Alkaloids	Spiked Conc. (ng/mL)	Dilution Factor	Measured Conc. (ng/mL)	Accuracy (%, RE)	Precision (%, RSD)
Corydaline (**1**)	369.45	10	33.07 ± 1.96	−10.5	5.9
		20	18.16 ± 0.80	−1.7	4.4
Tetrahydroberberine (**2**)	339.38	10	36.05 ± 1.27	6.2	3.5
		20	18.27 ± 1.02	7.7	5.6
Tetrahydropalmatine (**3**)	355.43	10	38.57 ± 2.32	8.5	6.0
		20	17.92 ± 0.15	0.8	0.8
Tetrahydrocolumbamine (**4**)	341.4	10	34.74 ± 0.66	0.0	3.7
		20	17.57 ± 0.65	−1.1	1.9
Tetrahydrocoptisine (**5**)	323.34	10	31.37 ± 1.25	−3.0	3.1
		20	15.67 ± 0.49	−3.0	4.0
Columbamine (**6**)	338.38	10	34.15 ± 2.06	0.9	6.0
		20	16.95 ± 0.49	0.2	2.9
Palmatine (**7**)	352.4	10	36.54 ± 1.73	3.7	4.7
		20	19.63 ± 0.17	11.4	0.8
Berberine (**8**)	336.36	10	31.44 ± 1.90	−6.5	6.0
		20	14.80 ± 0.70	−12.0	4.8
Epiberberine (**9**)	336.36	10	34.05 ± 2.08	1.2	6.1
		20	16.04 ± 0.65	−4.6	4.0
Coptisine (**10**)	1601.6	10	165.3 ± 6.02	3.2	3.6
		20	83.42 ± 1.79	4.2	2.2
Jatrorrhizine (**11**)	338.38	10	34.33 ± 0.64	0.4	2.6
		20	16.99 ± 0.45	1.4	1.9
Dehydrocorydaline (**12**)	366.43	10	34.71 ± 0.61	−4.0	8.4
		20	17.59 ± 1.48	−5.3	1.7
Oxoglaucine (**13**)	351.35	10	36.73 ± 1.92	4.5	5.2
		20	17.53 ± 0.84	−0.2	4.8
Protopine (**14**)	353.37	10	36.66 ± 2.20	3.8	6.0
		20	17.39 ± 0.81	−1.6	4.7

**Table 7:** Pharmacokinetic parameters of the fourteen alkaloids in male ICR mice after oral administration of *Corydalis yanhusuo* extract. (*n* = 8, mean ± SD).

No.	Parameters	C_max_ (ng/mL)	T_max_ (min)	AUC_0−t_ (ng min/mL)	AUC_0−∞_ (ng min/mL)	MRT_0−t_ (min)	T_1/2z_ (min)
**1**	Corydaline	53.27 ± 23.9	11.88 ± 2.59	4213.67 ± 1053.77	4219.32 ± 1052.51	115.09 ± 16.58	129.76 ± 53.45
**2**	Tetrahydroberberine	36.00 ± 23.1	10.00 ± 2.67	2017.5 ± 625.79	2020.81 ± 626	86.64 ± 22.68	90.31 ± 29.54
**3**	Tetrahydropalmatine	196.8 ± 116.3	17.50 ± 8.02	23,339.78 ± 7008.07	23,532.54 ± 7028.61	111.04 ± 17.06	62.11 ± 28.68
**4**	Tetrahydrocolumbamine	29.45 ± 22.39	35.63 ± 11.16	5330.56 ± 1151.48	5629.85 ± 1260.27	142.33 ± 9.13	94.02 ± 42.89
**5**	Tetrahydrocoptisine	65.86 ± 27.11	10.63 ± 1.77	6399.24 ± 1272.69	6402.31 ± 1274.6	123.42 ± 17.29	82.19 ± 30.62
**6**	Columbamine	1.66 ± 1.01	10.00 ± 2.67	492.33 ± 155.03	513.41 ± 155.25	327.8 ± 92.90	346.25 ± 148.93
**7**	Palmatine	14.33 ± 11.83	15.00 ± 6.55	2122.38 ± 549.93	2394.48 ± 518.15	169.29 ± 26.40	130.94 ± 28.77
**8**	Berberine	ND *	ND	ND	ND	ND	ND
**9**	Epiberberine	ND	ND	ND	ND	ND	ND
**10**	Coptisine	18.73 ± 13.31	28.13 ± 9.61	8039.17 ± 1924.04	8917.46 ± 1825.53	387.9 ± 90.88	432.93 ± 209.37
**11**	Jatrorrhizine	1.78 ± 0.57	15.63 ± 6.23	449.51 ± 160.24	461.64 ± 152.08	246.78 ± 59.02	196.09 ± 90.99
**12**	Dehydrocorydaline	8.50 ± 6.57	11.88 ± 7.53	2185.27 ± 1137.06	2222.64 ± 1125.92	163.18 ± 77.61	90.71 ± 56.48
**13**	Oxoglaucine	32.73 ± 21.2	15.00 ± 7.07	4797.27 ± 329.42	4831.29 ± 336.64	232.27 ± 33.42	202.93 ± 50.20
**14**	Protopine	26.43 ± 24.22	21.25 ± 9.54	3025.95 ± 736.76	3075.23 ± 706.99	163.5 ± 21.94	318.76 ± 251.55

* ND = Not detected within the linear range.

## Supplementary Materials

The following are available online, Figure S1: Product ion electrospray mass scan spectra of the 14 alkaloid and IS, Figure S2: Representative MRM chromatograms of (A) blank plasma, and (B) blank plasma spiked with the analytes at LLOQ and IS.
