# Supplementary material for: Development and Validation of a HPLC-ESI-MS/MS Method for Simultaneous Quantification of Fourteen Alkaloids in Mouse Plasma after Oral Administration of the Extract of Corydalis yanhusuo Tuber: Application to Pharmacokinetic Study
Source: Molecules. 2018 Mar 21;23(4):714. doi: 10.3390/molecules23040714 (PMC6017933; doi:10.3390/molecules23040714)
Supplement: Supplementary file 1 [file molecules-23-00714-s001.pdf]

(1) Corydaline

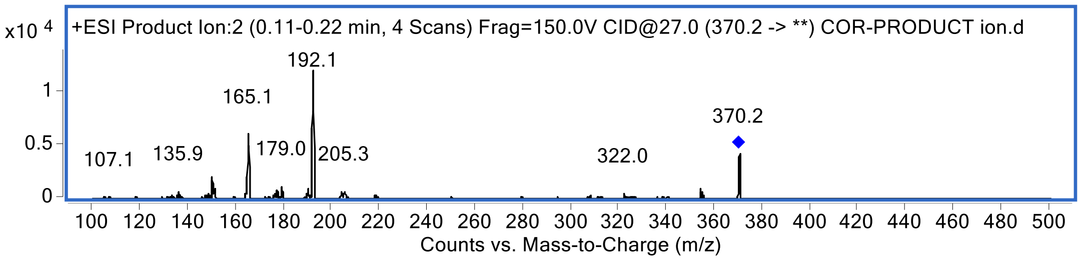

(2) Tetrahydroberberine

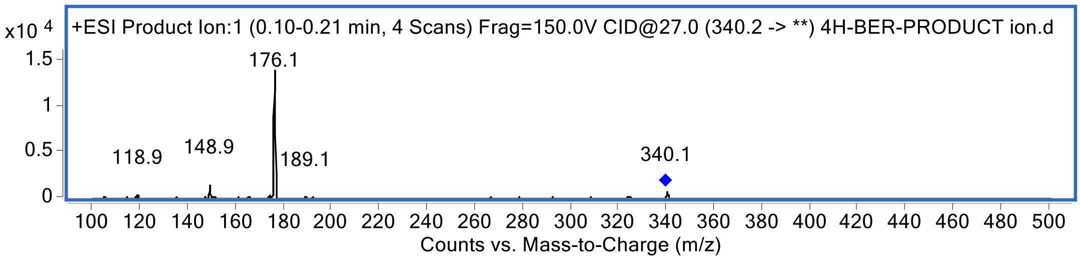

(3) Tetrahydropalmatine

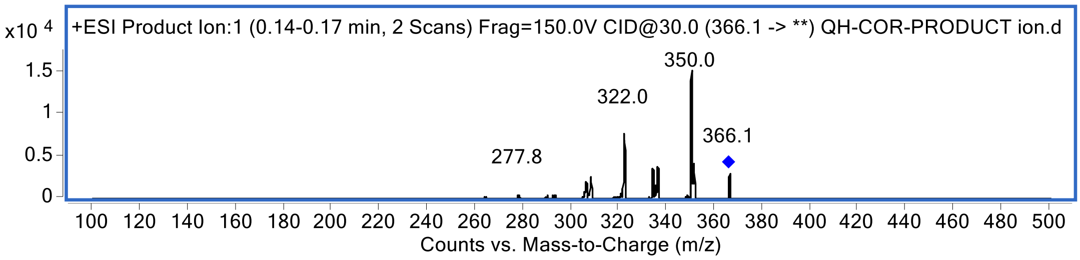

(4) Tetrahydrocolumbamine

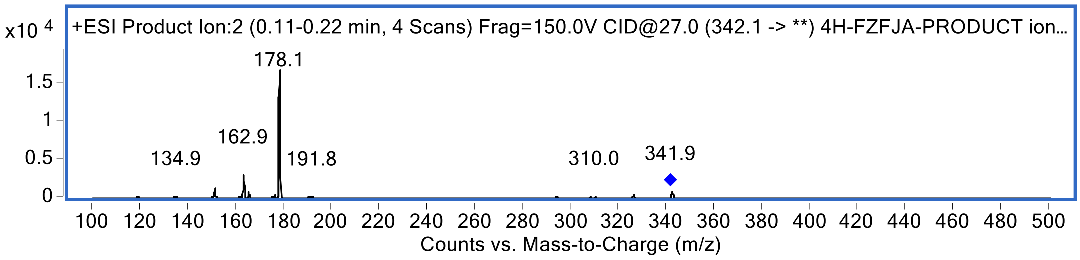

(5) Tetrahydrocoptisine

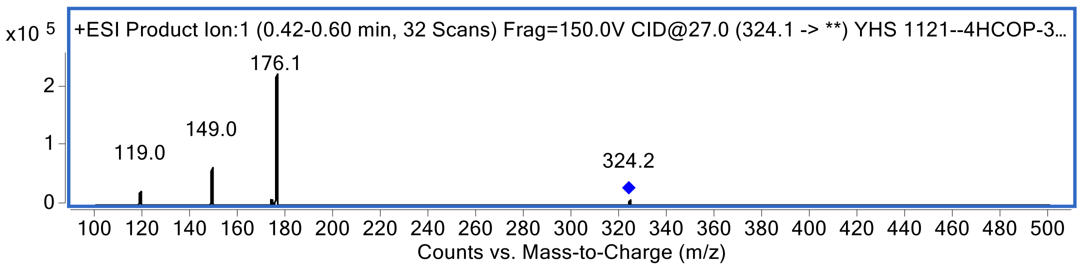

(6) Columbamine

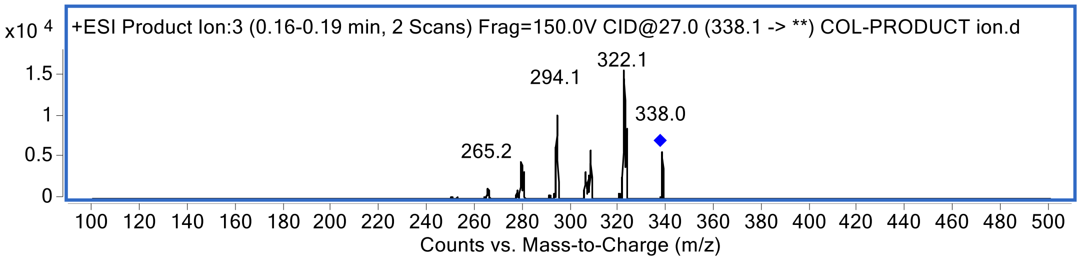

(7) Palmatine

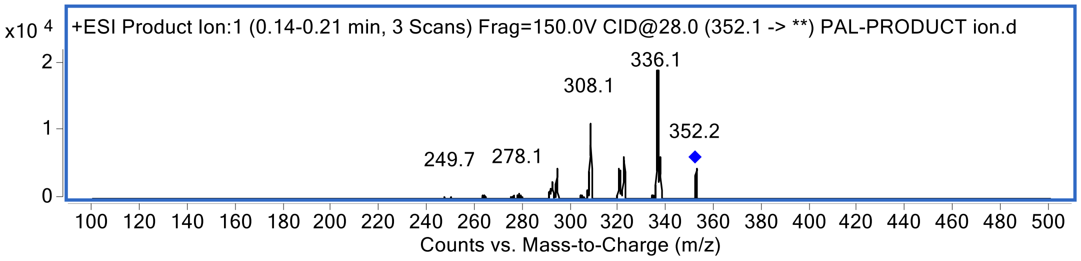

(8) Berberine

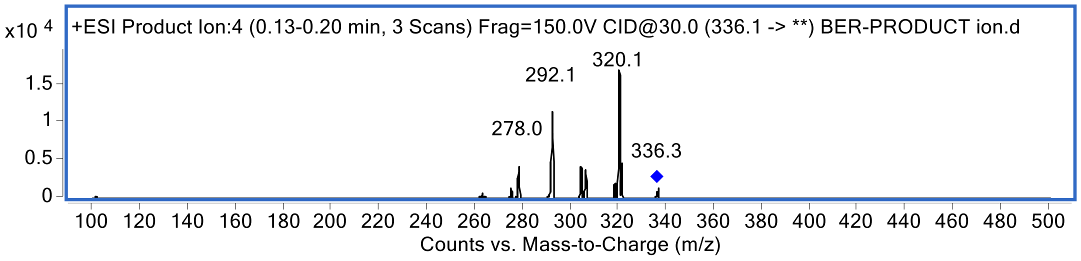

(9) Epiberberine

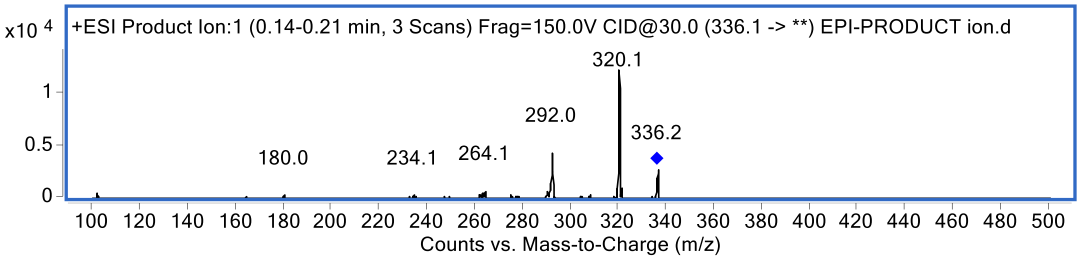

(10) Coptisine

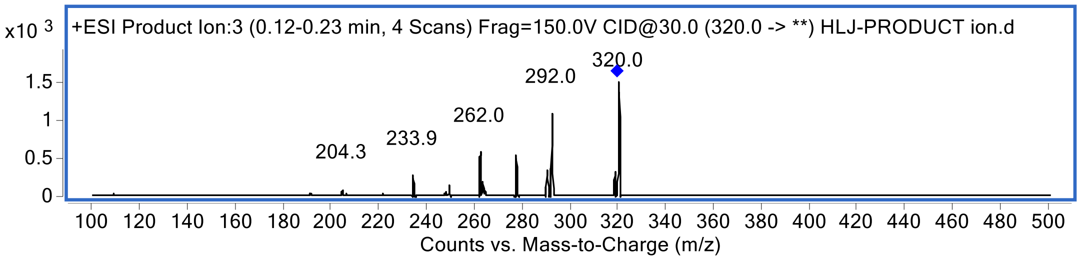

(11) Jatrorrhizine

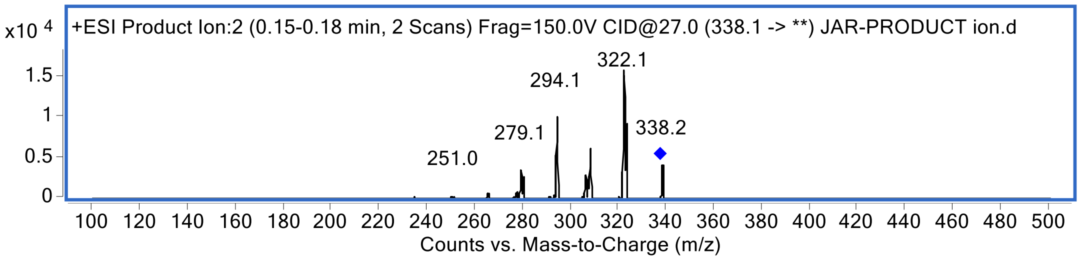

(12) Dehydrocorydaline

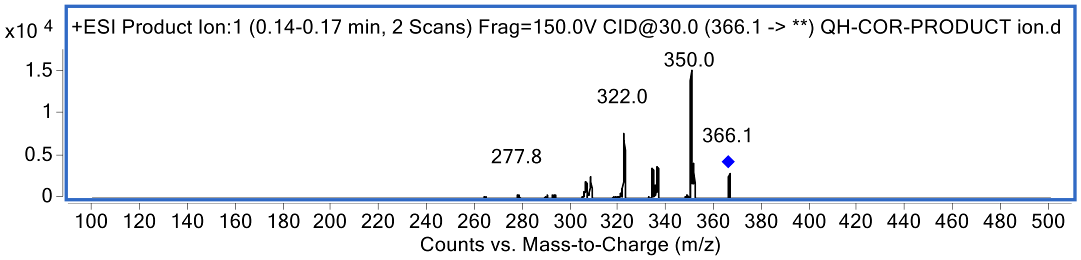

(13) Oxoglaucine

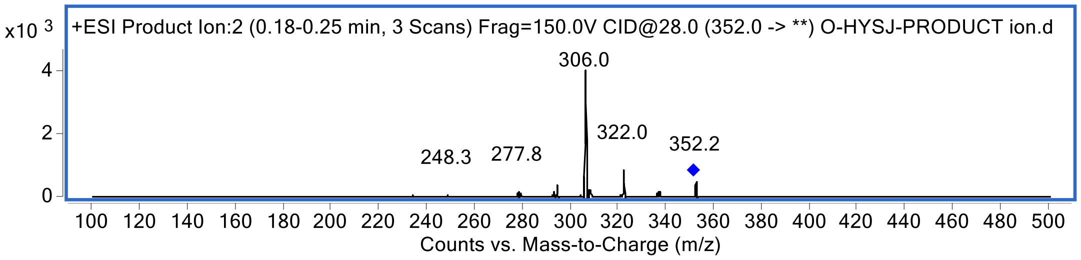

(14) Protopine

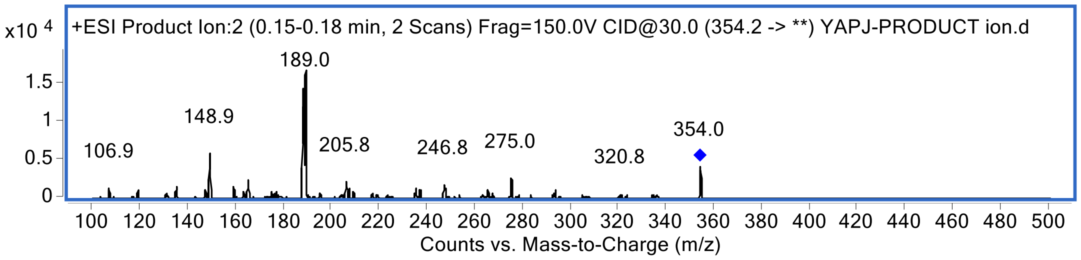

(IS) Nitidine chloride

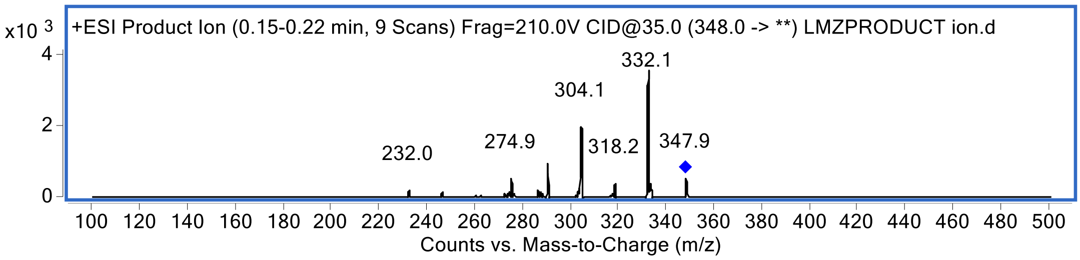

Supplementary Fig. S1 . Product ion electrospray mass scan spectra of the 14 alkaloid and IS.

A

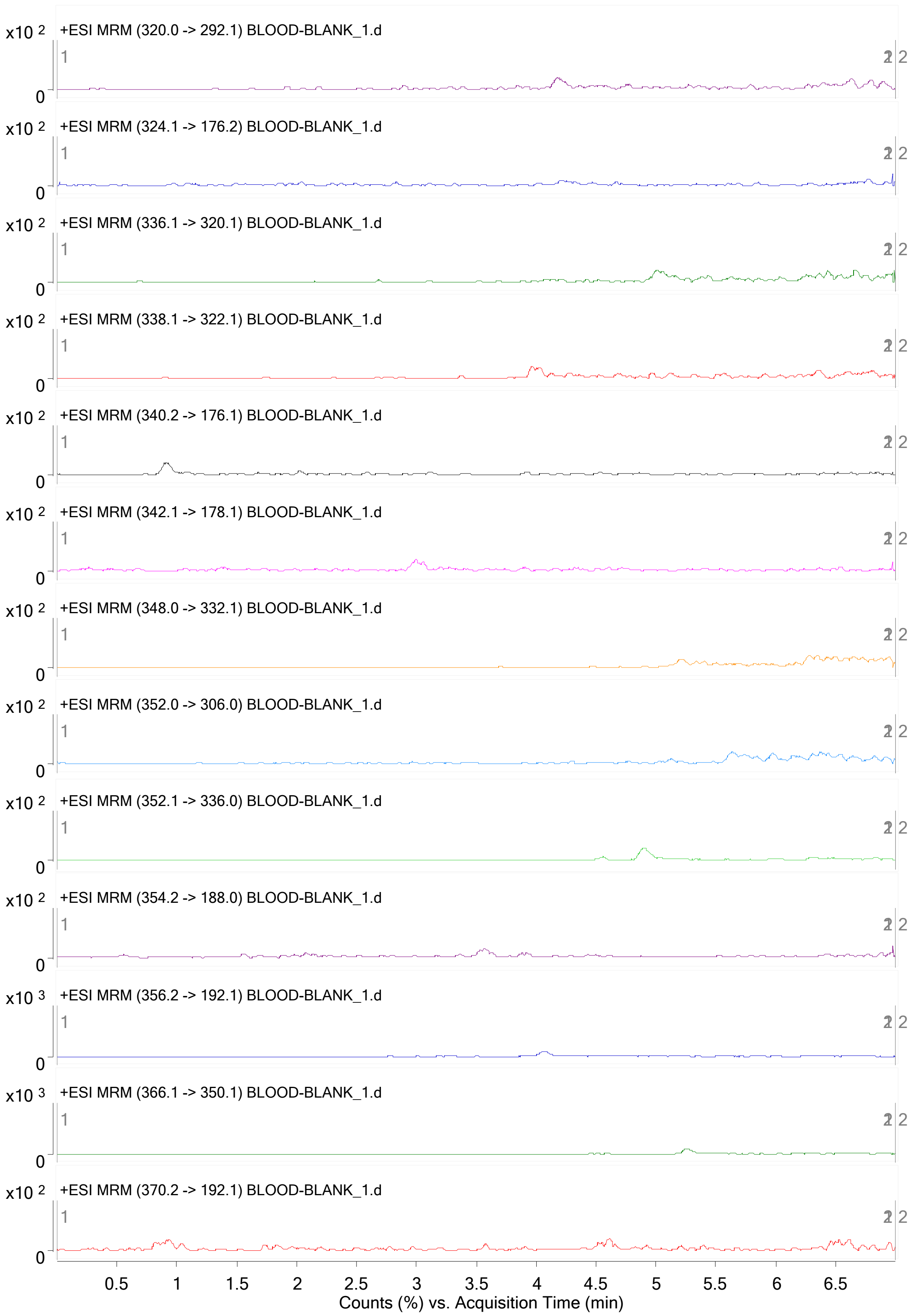

x10 2
+ESI MRM (320.0 -> 292.1) BLOOD-BLANK_1.d
x10 2
+ESI MRM (324.1 -> 176.2) BLOOD-BLANK_1.d
x10 2
+ESI MRM (336.1 -> 320.1) BLOOD-BLANK_1.d
x10 2
+ESI MRM (338.1 -> 322.1) BLOOD-BLANK_1.d
x10 2
+ESI MRM (340.2 -> 176.1) BLOOD-BLANK_1.d
x10 2
+ESI MRM (342.1 -> 178.1) BLOOD-BLANK_1.d
x10 2
+ESI MRM (348.0 -> 332.1) BLOOD-BLANK_1.d
x10 2
+ESI MRM (352.0 -> 306.0) BLOOD-BLANK_1.d
x10 2
+ESI MRM (352.1 -> 336.0) BLOOD-BLANK_1.d
x10 2
+ESI MRM (354.2 -> 188.0) BLOOD-BLANK_1.d
x10 3
+ESI MRM (356.2 -> 192.1) BLOOD-BLANK_1.d
x10 3
+ESI MRM (366.1 -> 350.1) BLOOD-BLANK_1.d
x10 2
+ESI MRM (370.2 -> 192.1) BLOOD-BLANK_1.d
0.5 1 1.5 2 2.5 3 3.5 4 4.5 5 5.5 6 6.5
Counts (%) vs. Acquisition Time (min)

B

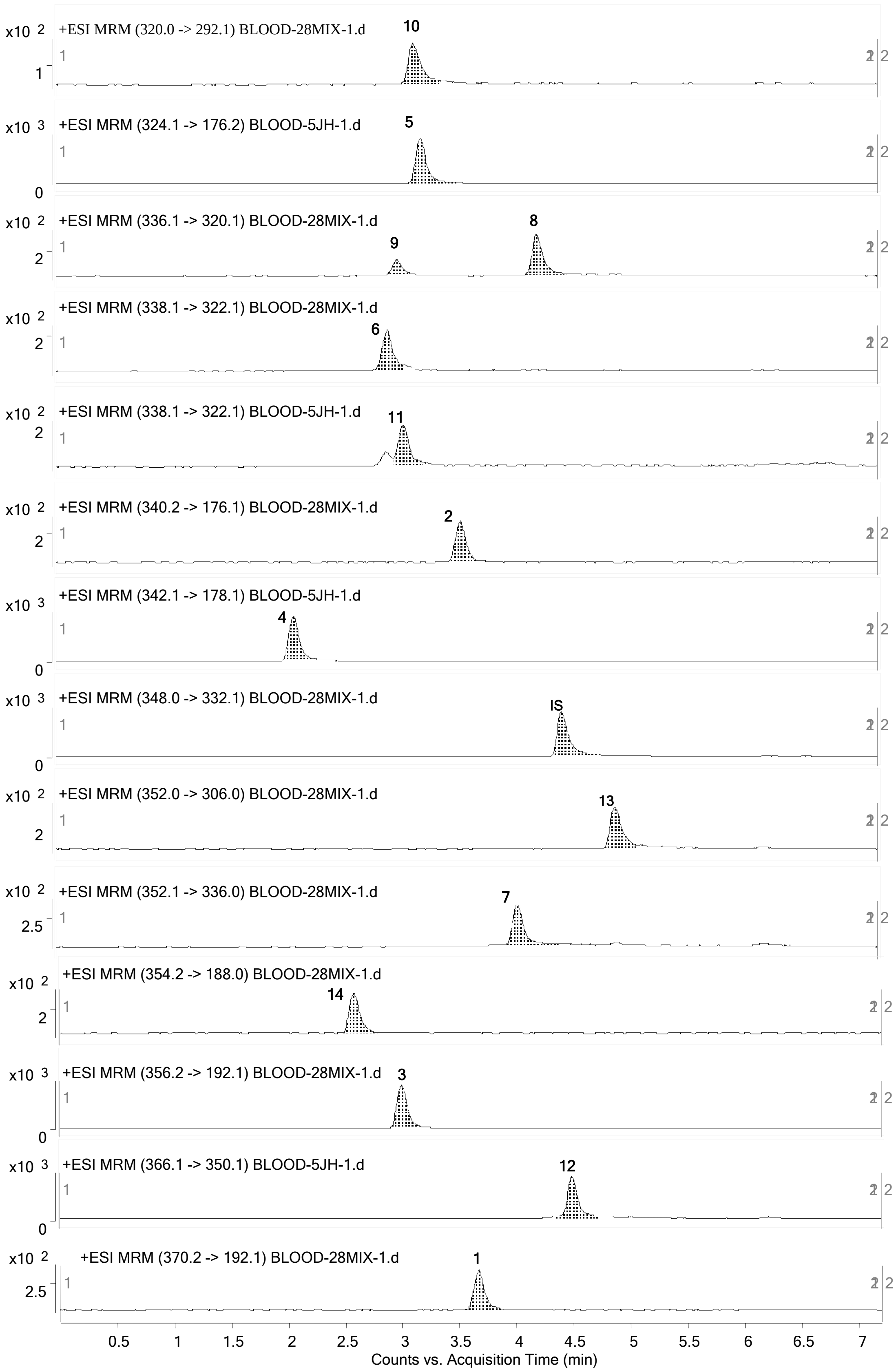

Supplementary Fig. S2 . Representative MRM chromatograms of (A) blank plasma, and (B) blank plasma spiked with the analytes at LLOQ and IS.
